# Antibacterial activity of ovatodiolide isolated from *Anisomeles indica* against *Helicobacter pylori*

**DOI:** 10.1038/s41598-019-40735-y

**Published:** 2019-03-12

**Authors:** Hsiu-Man Lien, Hui-Yu Wu, Chiu-Lien Hung, Chih-Jung Chen, Chia-Lin Wu, Kuan-Wen Chen, Chao-Lu Huang, Sheau-Jiun Chang, Chia-Chang Chen, Hwai-Jeng Lin, Chih-Ho Lai

**Affiliations:** 1 Research Institute of Biotechnology, Hungkuang University, Taichung, Taiwan; 2 Department of Microbiology and Immunology, Department of Biochemistry, Graduate Institute of Biomedical Sciences, College of Medicine, Chang Gung University, Taoyuan, Taiwan; 3 Targeted Drug and Delivery Technology Division, Biomedical Technology and Device Research Laboratories, Industrial Technology Research Institute, Hsinchu, Taiwan; 4 Department of Pediatrics, Department of Neurology, Molecular Infectious Disease Research Center, Chang Gung Memorial Hospital, Linkou, Taiwan; 5 Molecular Science Center, Genetics Generation Advancement, Taipei, Taiwan; 6 Department of Life Sciences, National Chung Hsing University, Taichung, Taiwan; 7 Department of Rehabilitation, Dachien General Hospital, Miaoli, Taiwan; 8 School of Management, Feng Chia University, Taichung, Taiwan; 9 Division of Gastroenterology and Hepatology, Department of Internal Medicine, School of Medicine, College of Medicine, Taipei Medical University, Taipei, Taiwan; 10 Division of Gastroenterology and Hepatology, Department of Internal Medicine, Shuang-Ho Hospital, New Taipei, Taiwan; 11 Department of Nursing, Asia University, Taichung, Taiwan; 12 School of Medicine, Department of Medical Research, China Medical University and Hospital, Taichung, Taiwan

## Abstract

*Helicobacter pylori* infection is associated with high incidence of gastric diseases. The extensive therapy of *H*. *pylori* infection with antibiotics has increased its resistance rates worldwide. Ovatodiolide, a pure constituent isolated from *Anisomeles indica*, has been demonstrated to possess bactericidal activity against *H*. *pylori*. In this study, ovatodiolide inhibited the growth of both *H*. *pylori* reference strain and clinical multidrug-resistant isolates. Docking analysis revealed that ovatodiolide fits into the hydrophobic pocket of a ribosomal protein, RpsB. Furthermore, ovatodiolide inhibited bacterial growth by reducing levels of RpsB, which plays a crucial role in protein translation. Our results demonstrate that ovatodiolide binds to a ribosomal protein and interferes with protein synthesis. This study provides evidence that ovatodiolide has the potential to be developed into a potent therapeutic agent for treating *H*. *pylori* infection.

## Introduction

*Helicobacter pylori* is a gram-negative species of bacteria that colonizes the gastric epithelium, causing chronic gastritis, peptic ulcer, gastric cancer, and mucosa-associated lymphoid tissue (MALT) lymphoma^1–3^. Persistent *H*. *pylori* infection in the human stomach leads to the secretion of several chemokines that induce chronic inflammation^4^. Studies have reported that eradication of *H*. *pylori* decreased the incidence of its associated gastrointestinal disorders^5,6^.

The standard methods for treating *H*. *pylori* infection are multidrug regimens that involve the combination of proton pump inhibitors and various types of antibiotics^7^. However, the extensive treatment of *H*. *pylori* infection with antibiotics has increased its resistance rates and has become a global health concern^8^. Most importantly, treatment failure rates are rising up to 20–40% due to the development of antimicrobial resistance^9^. Therefore, it is necessary to develop alternative therapeutic agents to treat *H*. *pylori* infection.

Several naturally derived products, including extracts of medicinal plants and isolated bioactive molecules, possess anti-*H*. *pylori* activity. These products appear effective with minimal adverse side effects^10^. In addition, many herbal remedies demonstrate gastroprotective properties and have been used to treat *H*. *pylori*-associated gastrointestinal disease^11^. Despite empirical demonstration of prominent inhibitory effects on *H*. *pylori*, mechanisms by which medicinal herbs and plant-derived products harbor anti-inflammatory and/or gastroprotective effects require further investigation.

*Anisomeles indica* (L) Kuntze (Labiatae) is a traditional Chinese herb called ‘yu-chen-tsao’ in Chinese. It has been demonstrated to possess anti-inflammatory activity^12^ and has been used to treat gastrointestinal diseases^13^. Ovatodiolide, a compound isolated from *A*. *indica*, has been reported to exhibit many biological functions, including anti-cancer^14–16^, anti-bacterial^17,18^, and anti-HIV activities^19^. Most importantly, our previous studies have demonstrated that ovatodiolide inhibited *H*. *pylori*-induced inflammation in gastric epithelial cells^17,20^. In this study, we further investigated the detailed mechanism of inhibition against *H*. *pylori* by ovatodiolide.

## Materials and Methods

### Chemicals and reagents

Dual-Luciferase Reporter Assay System and *E*. *coli* S30 Extract System were purchased from Promega (Madison, MA). Anti-RpsB antibody was purchased from MyBiosource (San Diego, California). Whole plant of *A*. *indica* was obtained from Yushen Co., Ltd (Taichung, Taiwan)^17^.

### Bacterial strains and culture

*H*. *pylori* 26695 (ATCC 700392), used as a reference strain was described previously^21^. Multidrug resistant (MDR)-*H*. *pylori* strains (v633 and v1354), which were clinical isolates and characterized as resistant to both metronidazole and clarithromycin^22^. All *H*. *pylori* strains were routinely cultured on Brucella blood agar plates (Becton Dickinson, Franklin Lakes, NJ) containing 10% sheep blood under 5% CO_2_ and 10% O_2_ conditions at 37 °C for 48 h.

### Preparation and characterization of ovatodiolide

The isolation of ovatodiolide from *A*. *indica* was described previously^20^. The purified ovatodiolide was confirmed by high-performance liquid chromatography (HPLC). The mobile phase consisted of acetonitrile and 0.1% trifluoroacetic acid (TFA) in water, 64:36 (UV detection at 265 nm).

### Determination of anti-*H*. *pylori* activity by ovatodiolide

Anti-*H*. *pylori* activities of ovatodiolide were determined by disc agar diffusion method as described previously^17^. Briefly, *H*. *pylori* suspension [1 × 10^8^ colony forming units (CFU)] was spread on Brucella blood agar plates containing 10% sheep blood. Different concentrations of ovatodiolide were added to the paper discs. The plates were cultured in microaerophilic condition for 48 h and the inhibition zone was determined in diameter.

### Transcription/translation assay and luminescence read out

Transcription/translation assay was performed as described previously^23,24^. Various concentrations of ovatodiolide, kanamycin, erythromycin, and negative controls (0.4% DMSO) were mixed with diluted *E*. *coli* S30 extract. This mixture was incubated for 10 minutes at 25 °C. Diluted premix reagent, consisting of S30 premix without amino acid; complete amino acid; H_2_O and 1 µg pGL3 plasmid DNA, were mixed and incubated for 2 h at 25 °C. After incubation, luciferase activity was detected for each sample. Luciferase assays were performed with the Dual-Luciferase Reporter Assay System (Promega) using a microplate luminometer (Biotek, Winooski, VT).

### Structural modeling and docking

The RpsB model was prepared with BIOVIA Discovery Studio software (Dassault Systèmes BIOVIA, Discovery Studio Modeling Environment, Release 2018, San Diego: Dassault Systèmes, 2016)^25^, employing multiple ribosomal subunit proteins from *E*. *coli* and *Thermus thermophilus* (Protein Data Bank Codes: 4TOI, 2E5L, 4YHH, 4 × 62, and 1F1G). The binding sites were defined using the eraser algorithm. The docking protocol was utilized Flexible Docking which initiated ligand replacement by LibDock and refined the docking poses using CDOCKER^26^. The scoring function are reported as the negative of the energy values by CDOCKER interaction scores. All initial binding sites and docking analyses employed CDOCKER. Structural figures were also generated using BIOVIA Discovery Studio software^25^.

### Western blot analysis

The level of RpsB expression was determined by western blot analysis. *E*. *coli* were treated different concentrations of ovatodiolide for 6 h. The cell lysates were prepared and subjected to 10% sodium dodecyl sulphate-polyacrylamide gel electrophoresis (SDS-PAGE) then transferred onto polyvinylidene difluoride (PVDF) membrane (Pall, East Hills, NY) for western blot analysis. RpsB was probed with rabbit anti-RpsB antibody. The proteins of interest were visualized using enhanced chemiluminescence reagents (GE Healthcare, Buckinghamshire, UK) and were detected by Azure C400 biosystems. The total cell lysates were subjected to SDS-PAGE and determined by staining with Ponceau S (Sigma-Aldrich, St. Louis, MO).

### Statistical analysis

Statistical significance analysis was performed using Student’s *t*-test; a *P* value < 0.05 was considered significant.

## Results

### Ovatodiolide inhibited the growth of *H*. *pylori*

Various concentrations of ovatodiolide (0–20 µM) were used against a *H*. *pylori* reference strain through a disc agar diffusion assay. At 10 µM and 20 µM, the inhibition zones were measured to be 10 ± 0.5 mm and 19 ± 0 mm, respectively (Table 1). We then explored whether ovatodiolide possessed bactericidal activity for both reference and clinical MDR-*H*. *pylori* strains. As shown in Table 2, MDR-*H*. *pylori* strains (v633 and v1354) showed resistant to erythromycin. However, the minimum bactericidal concentration (MBC) of ovatodiolide for reference and MDR-*H*. *pylori* are 200 µM and 100 µM, respectively. These results indicate that ovatodiolide exerted bactericidal activity against both *H*. *pylori* reference and MDR strains.

**Table 1:** Growth inhibition of *H*. *pylori* reference strain by ovatodiolide.

Ovatodiolide (μM)	Inhibition zone (mm)^†^
20.0	19.0 ± 0
10.0	10.0 ± 0.5
5.0	—^‡^
2.5	—
1.25	—
0	—

^†^Data are shown as means ± SD.

^‡^Without inhibition zone.

**Table 2:** Minimum bactericidal concentration of ovatodiolide against *H*. *pylori* reference and MDR strains.

*H*. *pylori*^†^	Ovatodiolide (μM)	Kanamycin (μM)	Erythromycin (μM)
Reference strain 26695	200	100	50
v633	100	100	R^‡^
v1354	100	100	R

^†^Hp 26695 was a reference strain. Strains v663 and v1354 were MDR clinical isolates, which showed resistant to metronidazole and clarithromycin.

^‡^Resistant to the antibiotic.

### Ovatodiolide inhibited protein synthesis

**Figure 1:**
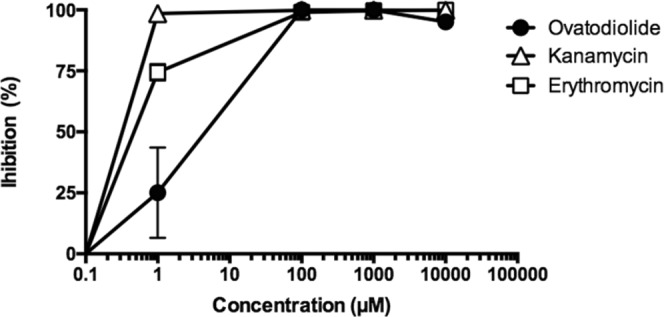
Effect of ovatodiolide on protein synthesis by *in vitro* transcription/translation assay. Treatments of ovatodiolide were administered at the concentrations of 0.1–10000 µM. Standard antibiotics, kanamycin and erythromycin, were used as positive controls. Results are means ± SD from 3 independent experiments.

Because the structure of ovatodiolide is similar to that of macrolides, we then investigated whether ovatodiolide acts against *H*. *pylori* by interrupting the translation process. Protein synthesis activity was determined using an *in vitro* transcription/translation system. As shown in Fig. 1, the concentration of ovatodiolide that inhibited 50% of protein synthesis was 2.8 µM. The other two antibiotics, kanamycin and erythromycin, which are protein synthesis inhibitors, inhibited 50% protein synthesis at 0.21 µM and 0.45 µM, respectively. These suggest that ovatodiolide inhibited bacterial growth by interfering with protein synthesis.

### Ovatodiolide functions against ribosomal protein

**Figure 2:**
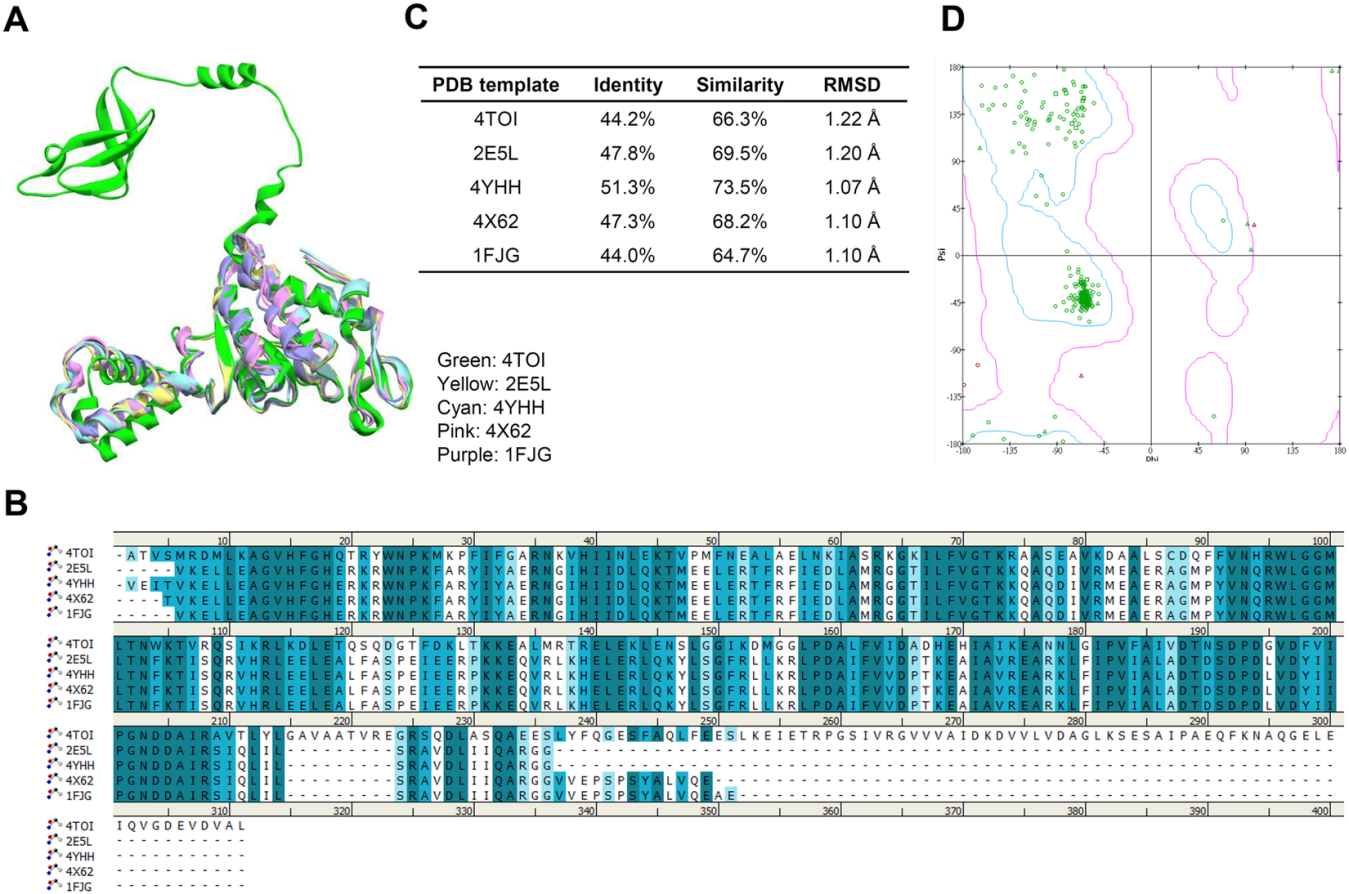
Molecular modeling of RpsB. (**A**) Superimposed RpsB models. (**B**) Multiple sequence alignments of ribosomal subunits. (**C**) Summary of sequence identify, similarity, and RMSD of structure models. (**D**) Ramachandran plot for the RpsB model.

**Figure 3:**
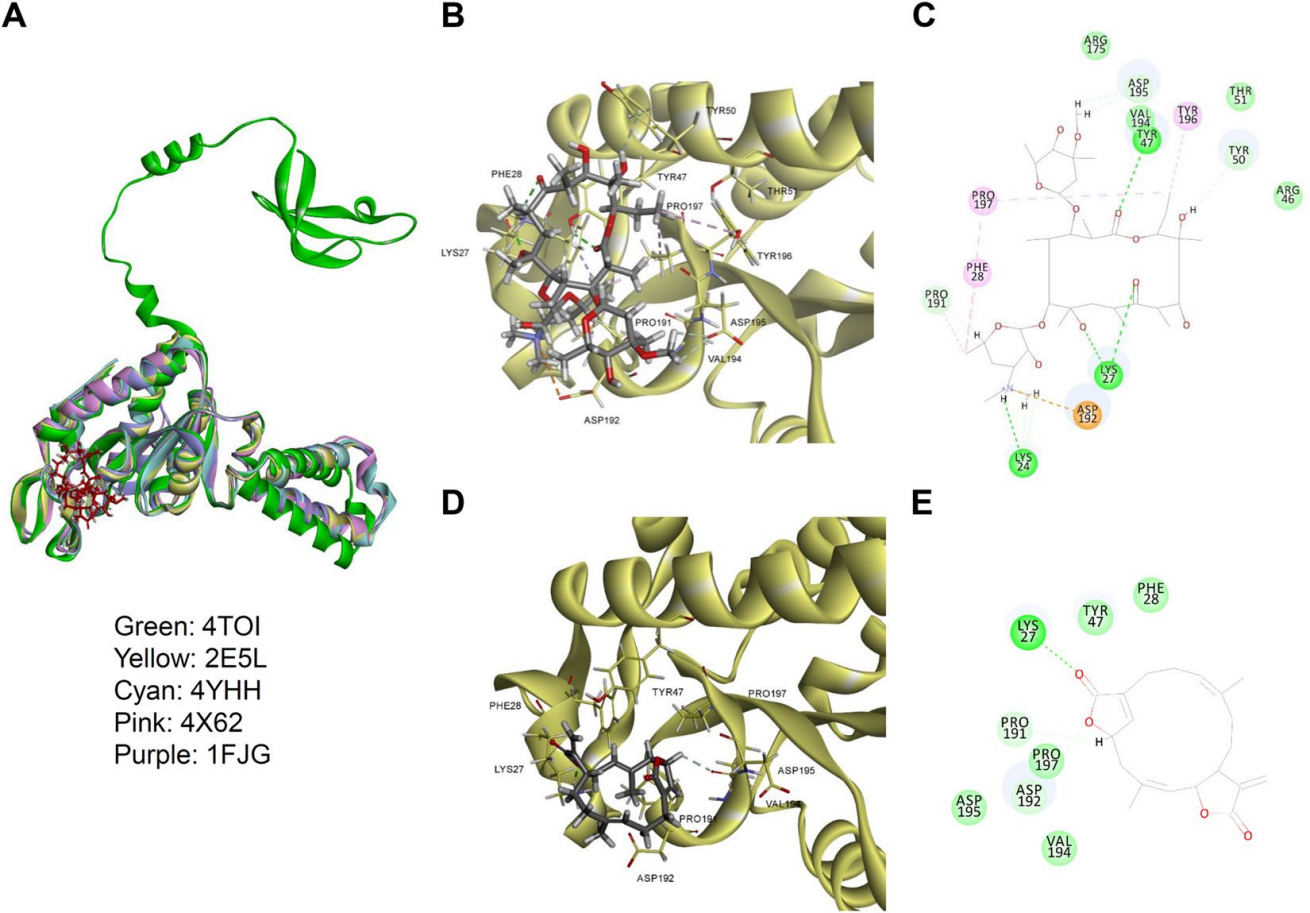
Molecular docking model for the interaction of ovatodiolide/erythromycin with RpsB. (**A**) Superimposed docking models for ovatodiolide/erythromycin-RpsB. Ovatodiolide and erythromycin are shown in stick-form. RpsB subunit (PDB code: 4TOI, 2E5L, 4YHH, 4 × 62, and 1F1G) are shown as cartoons. 3D molecular interactions are depicted for (**B**) erythromycin-RpsB, and (**D**) ovatodiolide-RpsB, respectively. Ovatodiolide and erythromycin are shown in bold stick form, and potential binding sites are shown in light stick form. 2D molecular interactions are depicted for (**C**) erythromycin-RpsB, and (**E**) ovatodiolide-RpsB. Hydrogen-bond interactions are shown as green dashed lines. Van der Walls interactions are shown as light green dashed lines. Alkyl interaction is shown as a pink dashed line. Docking analyses used BIOVIA Discovery Studio as described in the Methods section.

Multiple ribosomal subunits were used as template RpsB to build the RpsB model (Fig. 2A). Template sequence alignments were highly conserved and showed identities of 44.0–51.3% (Fig. 2B,C). RMSD for C-alpha atom backbones to reference were 1.07–1.22 Å (Fig. 2C). Model quality was represented via a Ramachandran plot (Fig. 2D). Since the chemical structure of ovatodiolide resembles that of macrolides (Fig. 3A), ovatodiolide may inhibit ribosomal proteins in a manner similar to erythromycin. The docking model suggested by the Flexible Docking Program (BIOVIA Discovery Studio) indicated that ovatodiolide occupied the hydrophobic groove of RpsB. The superimpose of docking model with employing multiple ribosomal subunit proteins (Protein Data Bank Codes: 4TOI, 2E5L, 4YHH, 4 × 62, and 1F1G) exhibited that ovatodiolide was occupied the similar binding site of RpsB (Fig. 3A). Amino acids in RpsB (Lys27, and Tyr47) were observed to directly contact erythromycin (Fig. 3B,C). In addition, ovatodiolide interacted with Lys27 and Val194 via hydrogen-bonding with 1.88 Å and 2.54 Å, and with Pro191 and Pro197 through Van der Walls forces (Fig. 3D,E). Together, the results indicate that ovatodiolide potentially interacts with RpsB.

**Figure 4:**
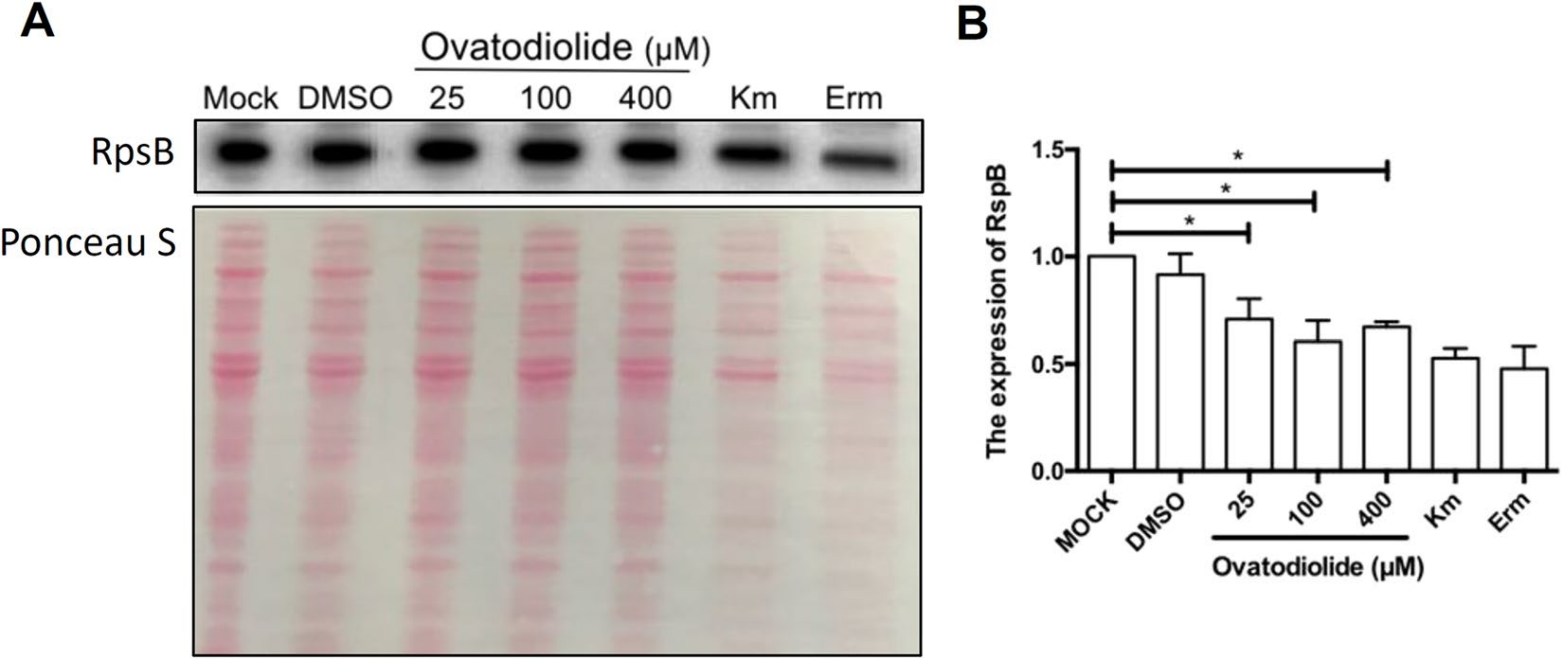
Effect of ovatodiolide on RpsB expression. (**A**) Bacteria were treated with various concentrations of ovatodiolide (25, 100, and 400 μM) for 6 h. The expression levels of RpsB were determined through western blot analysis (upper panel). The protein loading control of total cell lysates were subjected to SDS-PAGE and determined by staining with Ponceau S (lower panel). (**B**) RpsB expression was determined by densitometric analysis. ^*^P < 0.05 was considered statistically significant. Standard antibiotics, kanamycin (Km) and erythromycin (Erm), were used as positive controls. Results are means ± SD from 3 independent experiments.

We then explored whether ovatodiolide inhibited ribosomal proteins and obstructed protein synthesis. After treatment with ovatodiolide, RpsB expression was detected through western blot analysis. As shown in Fig. 4, the protein expression level of RpsB decreased upon ovatodiolide treatment. These results demonstrate that ovatodiolide fits into a ribosomal protein and may interfere with protein translation.

## Discussion

*H*. *pylori* colonizes the human stomach and causes several gastrointestinal diseases, including gastritis, peptic ulcer, and gastric adenocarcinoma^27^. Antimicrobial agents are the most effective for eradicating *H*. *pylori* infection, particularly through a triple therapy regimen consisting of a proton-pump inhibitor, amoxicillin, and clarithromycin^28^. However, resistance rates have elevated with the use of antimicrobial agents over time^8^. Therefore, it is necessary to discover potent agents for treating *H*. *pylori* infection. Accordingly, medicinal herb and plant derived-products with predominant effectiveness and low adverse effects are deserved to be explored.

*A*. *indica* extracts possess the ability to ameliorate inflammation^12^. Ovatodiolide, an important constituent of *A*. *indica*, has been found to exbibit activity not only against viruses^19^, but against bacteria as well^18^. Our previous study reported that ovatodiolide isolated from *A*. *indica* inhibited *H*. *pylori* growth^20^. We then demonstrated that *A*. *indica* contained a vast amount of ovatodiolide, which exerted the inhibitory effect on *H*. *pylori*-induced inflammation^17^. This study further suggested that ovatodiolide may exert bactericidal function by interfering with protein synthesis. Although these findings indicate that ovatodiolide is worth developed into a potential agent against *H*. *pylori* growth, long-term *in vivo* effectiveness and toxicity are warranted to be assessed.

The structure of ovatodiolide resembles that of erythromycin, which may explain its ability to inhibit bacterial protein synthesis. Docking analysis indicated that ovatodiolide and macrolides interact similarly with ribosomal proteins (Fig. 3A). Multiple sequence alignments showed six highly conserved regions (Fig. S2). Docking energy calculations from RpsB-ERY/OVT model results indicated that regions 1 and 6 are potential binding sites (Supplementary Table S1). Furthermore, to address an adequate docking pose, the complex structure of RpsB from *Thermus thermophiles* (PDB code: 4 × 62) was used to analyze the potential docking pose and potential interaction regions. The composition of complex structure includes 30 s ribosomal subunit and DNA sequence, the potential interaction region was located in region 1 of the docking pose (Suppl Figs S2B and S3). Interaction energies for RpsB-ERY and RpsB-OVT models were estimated by CDOCKER as −81.998 and −39.624 Kcal/mol, respectively. Moreover, ovatodiolide exerted activity against ribosomal subunit protein, RpsB (Fig. 4). These results suggest that the direct binding of ovatodiolide to a ribosomal subunit protein inhibited protein synthesis.

Emergence of MDR-*H*. *pylori* is a worldwide health concern. With increasing incidence of antimicrobial resistance, failure rates are sustainably rising^7^. Our previous study indicated that MDR-*H*. *pylori* strains (v633 and v1354) presented resistance to macrolides^22^. In this study, we showed that ovatodiolide inhibited both *H*. *pylori* reference and MDR strains (Table 2). To address the problem with the rising incidence of antimicrobial resistance, ovatodiolide may be an alternative agent for the therapy of *H*. *pylori* infection, particularly in patients infected with MDR strains.

In summary, this study reported that ovatodiolide isolated from *A*. *indica* exhibited inhibitory effects against *H*. *pylori* growth. We also demonstrated that ovatodiolide not only suppressed the growth of reference *H*. *pylori*, but also of MDR strains. In particular, the potential mechanism for the bactericidal activity of ovatodiolide may involve the inhibition of ribosomal translation. Therefore, ovatodiolide may have the potential to be developed into a new therapeutic agent against *H*. *pylori* infection.

## Supplementary information

Supplementary Information
