# Supplementary material for: Antibacterial activity of ovatodiolide isolated from Anisomeles indica against Helicobacter pylori
Source: Sci Rep. 2019 Mar 12;9:4205. doi: 10.1038/s41598-019-40735-y (PMC6414523; doi:10.1038/s41598-019-40735-y)
Supplement: Supplementary file 1 — Supplementary Information [file 41598_2019_40735_MOESM1_ESM.pdf]

Antibacterial activity of ovatodiolide isolated from *Anisomeles indica* against *Helicobacter pylori*

Hsiu-Man Lien[1], Hui-Yu Wu[2†], Chiu-Lien Hung[3†], Chih-Jung Chen[4], Chia-Lin Wu[2,4], Kuan-Wen Chen[5], Chao-Lu Huang[6], Sheau-Jiun Chang[7], Chia-Chang Chen[8], Hwai-Jeng Lin[9,10]*, Chih-Ho Lai[2,4,11,12]*

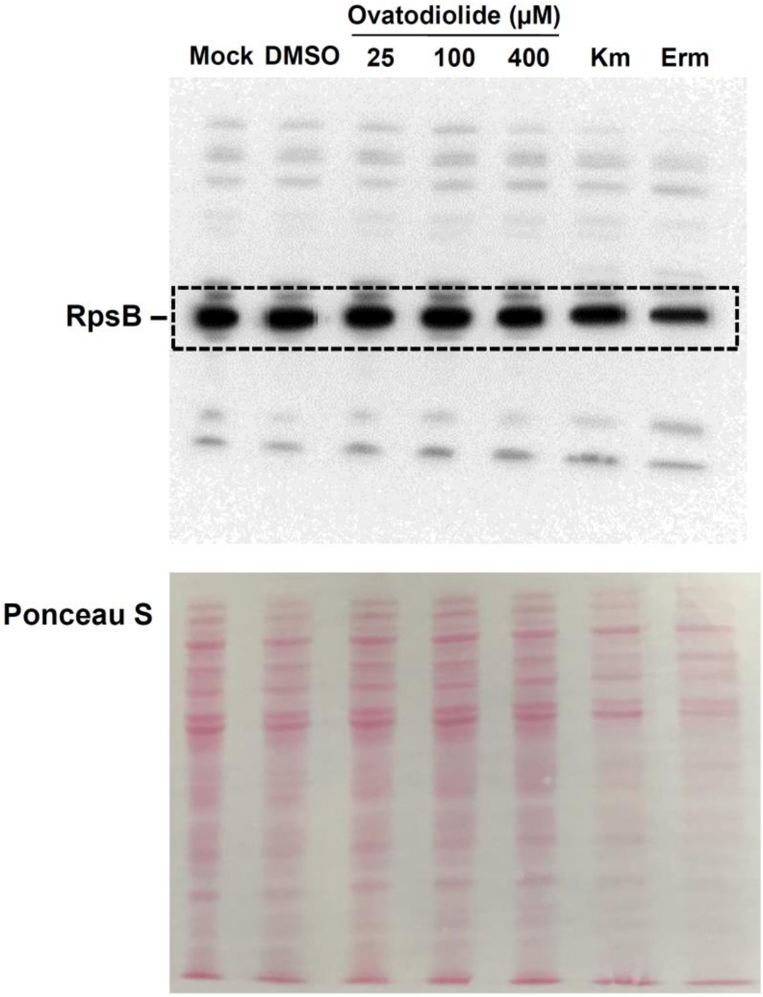

Supplementary Figure S1. Full uncropped images for Figure 4A.

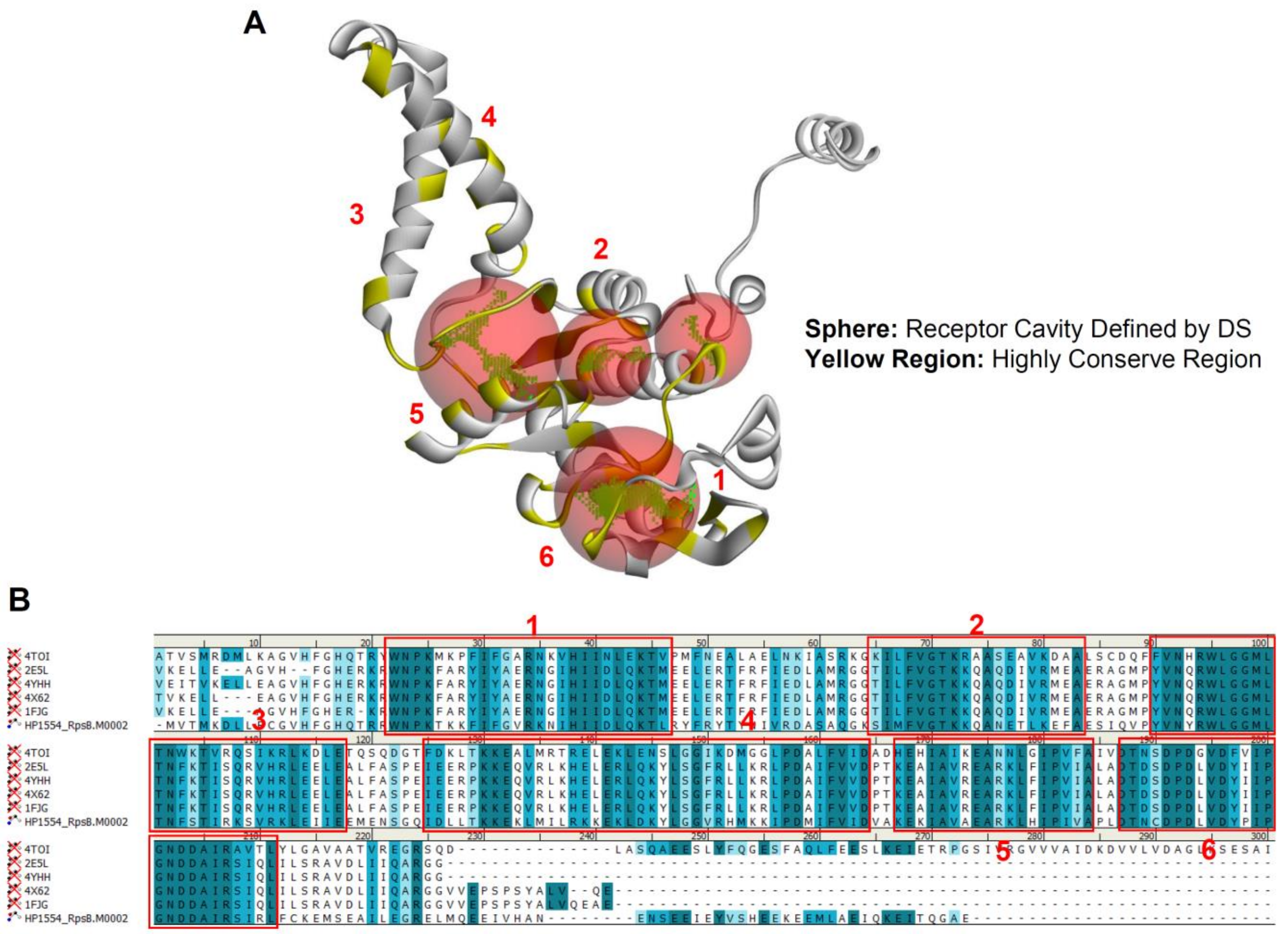

Supplementary Figure S2. Predicted binding sites for the RpsB model. (A) Highly conserved regions are shown in yellow and predicted receptor cavities are shown as red spheres. (B) Predicted receptor cavities are defined by sequences highlighted in red boxes and labeled for regions 1-6. BIOVIA Discovery Studio software was used for the analysis.

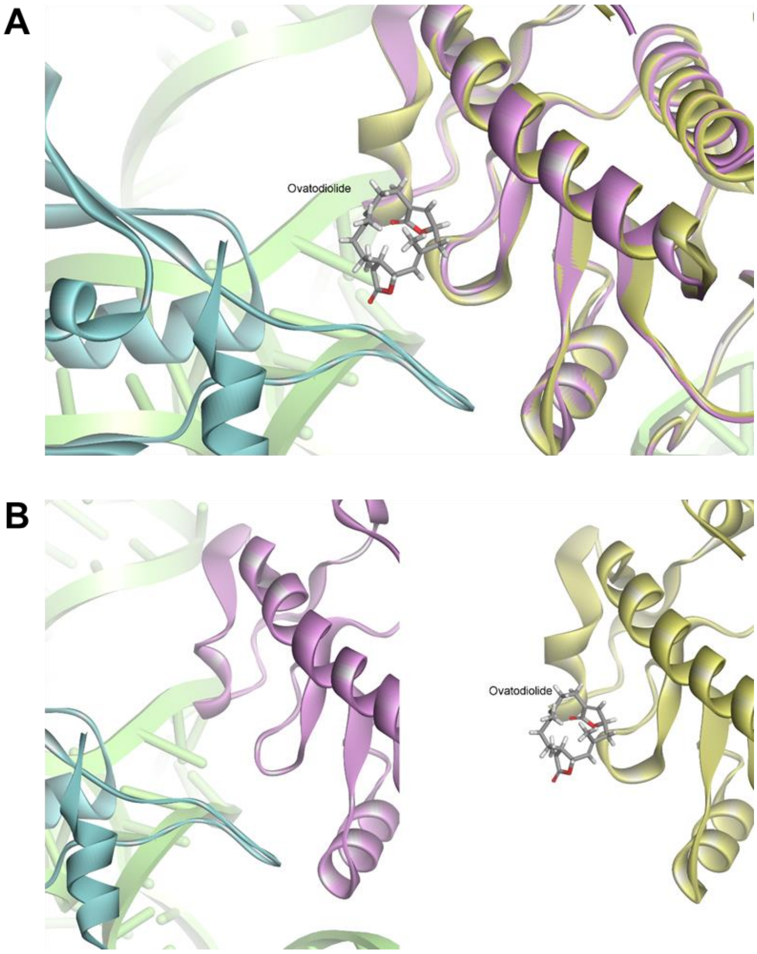

Supplementary Figure S3. The superimpose of 30S ribosomal complex structure and RpsB. (A) The docking model of partial 30S ribosomal complex structure and *H. pylori* RpsB. The model of *H. pylori* RpsB with ovatodiolide is shown in yellow, the structure of *Thermus thermophiles* RpsB is shown in magenta (PDB code: 4X62), and the structure of 30S ribosomal protein S8 structure is shown in cyan. (B) Left panel: The potential docking pose is presented within the view of the structure of *Thermus thermophiles* RpsB (magenta) and 30S ribosomal protein S8 structure (cyan). Right panel: The ovatodiolide (ball and stick) is docked adequately in potential docking region.

Supplementary Table S1. The docking results are summarized by the energy calculations

	CDOCKER interaction energy	Binding free energy
Hp RpsB modeling structure	25.4510	-39.6240
E. coli RpsB<br>(PDB code: 4TOI)	21.6958	-55.3462
Thermus thermophiles RpsB<br>(PDB code: 4X62)	21.6641	-30.6514

*Energy unit: kcal/mol
